# PROMOTION OF CULTURAL GENERATIVITY TO SUPPORT SUCCESSFUL AGING IN ALASKA NATIVE COMMUNITIES

**DOI:** 10.1093/geroni/igz038.3059

**Published:** 2019-11-08

**Authors:** Steffi M Kim, Jordan P Lewis

**Affiliations:** University of Alaska Anchorage, Anchorage, Alaska, United States

## Abstract

The psychological construct of generativity encompasses a person’s motivation to leave a legacy for future generations by investing in acts that will outlive the self (Ericson, 1950). Lewis and Allen (2017) outlined the importance of generativity within AN cultures (caring for the future of our youth) and expanded Erikson’s western-based definition by adding the indigenous cultural generativity component to describe and incorporate Alaska Native specific cultural elements. These culturally based elements involve giving back to family and community including guidance and teaching of future generations (Lewis & Allen, 2017). Generativity within western societies accentuates independent achievements and successes more than the notion to care for future generations. This study is part of a larger community-based, exploratory, study between researchers and communities to explore successful aging that included 42 Alaska Native Elders in the Norton-Sound subregion, 21 Alaska Native Elders from the Aleutian Pribilof Islands, and 26 Elders from the Bristol Bay region. Qualitative interviews explored the participant’s life, influences on aging well, and their aging process. Thematic analysis was used to investigate the impact of generativity on successful aging was used to establish codes and main themes based on the three different cultural regions of Alaska. The findings suggest that generativity promotes successful aging. Elders who live in communities that promote community engagement, support family and school activities, and maintain and/or revitalize culture and traditions reported increased emotional well-being. Results can guide communities to incorporate or support cultural activities that promote generative activities and meaningful engagement which fosters successful aging.

